# Supplementary material for: Stability of Phenols, Antioxidant Capacity and Grain Yield of Six Rice Genotypes
Source: Plants (Basel). 2023 Jul 27;12(15):2787. doi: 10.3390/plants12152787 (PMC10421503; doi:10.3390/plants12152787)
Supplement: Supplementary file 1 [file plants-12-02787-s001.zip › plants-2385314-supplementary.pdf]

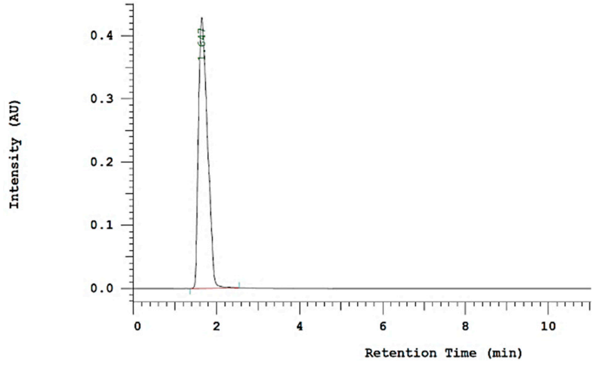

Figure S1. HPLC chromatogram of ferulic acid.

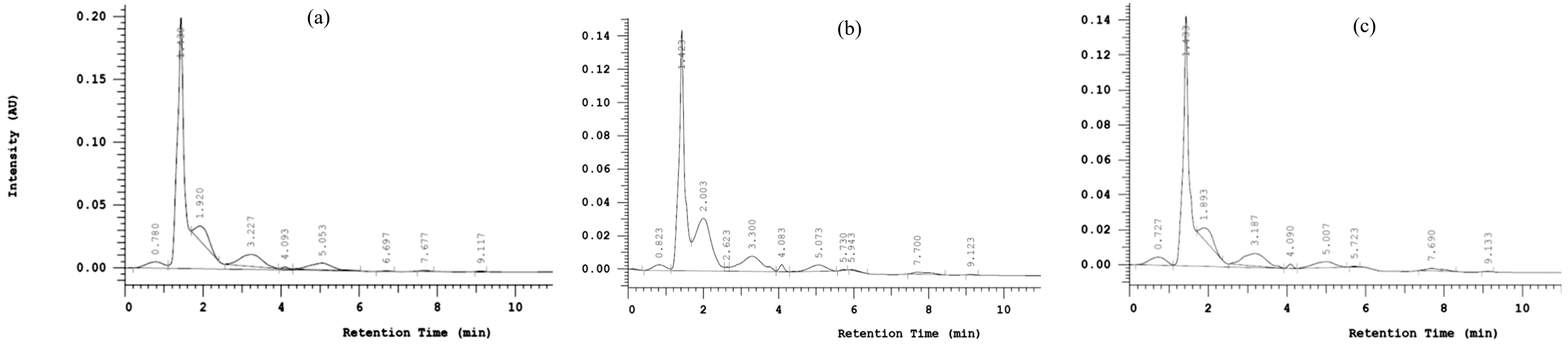

Figure S2 chromatogram of Hom Nang Nual growing in Bangkok (a), Trat (b) and Sakon Nakhon (c) province.

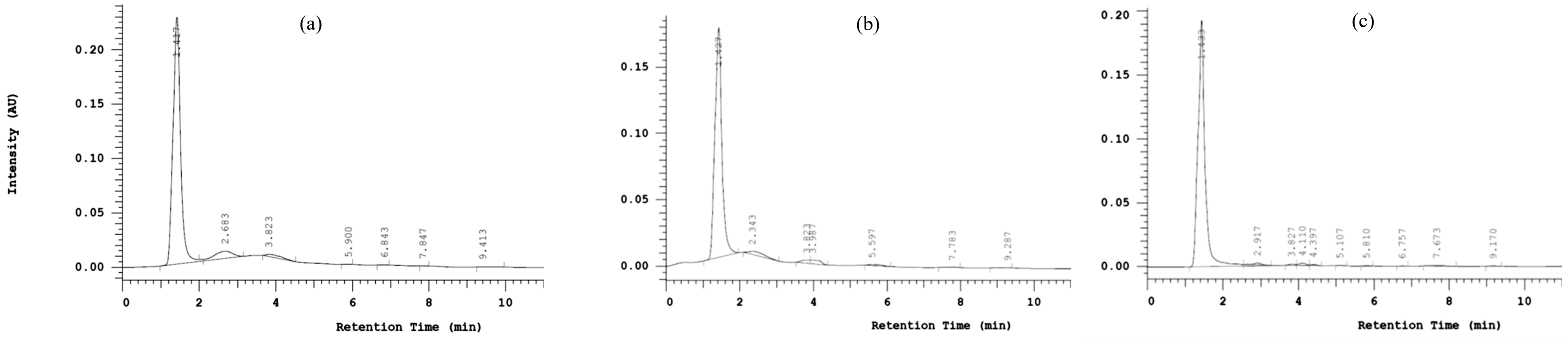

Figure S3 chromatogram of Mali Nil Boran growing in Bangkok (a), Trat (b) and Sakon Nakhon (c) province.

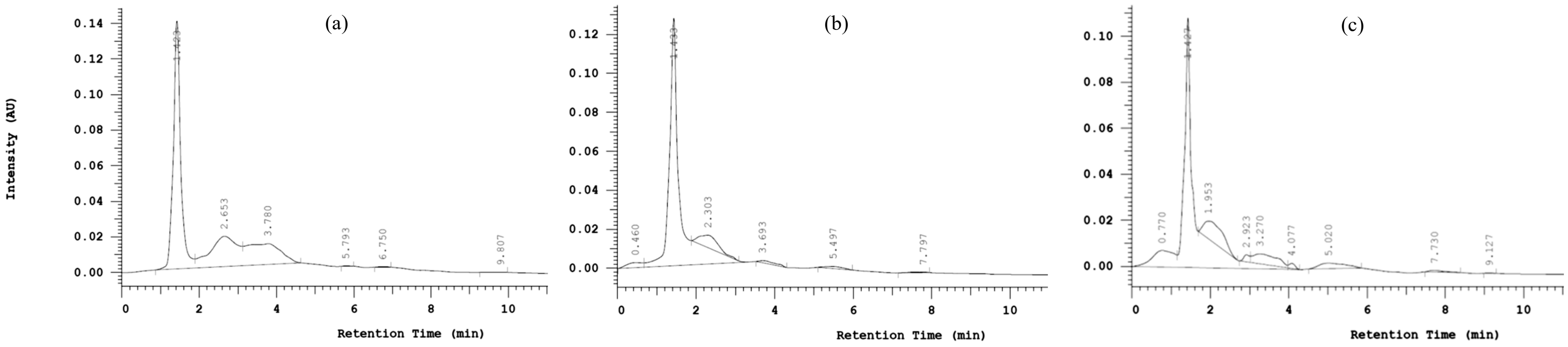

Figure S4 chromatogram of Lhueang Thong growing in Bangkok (a), Trat (b) and Sakon Nakhon (c) province.

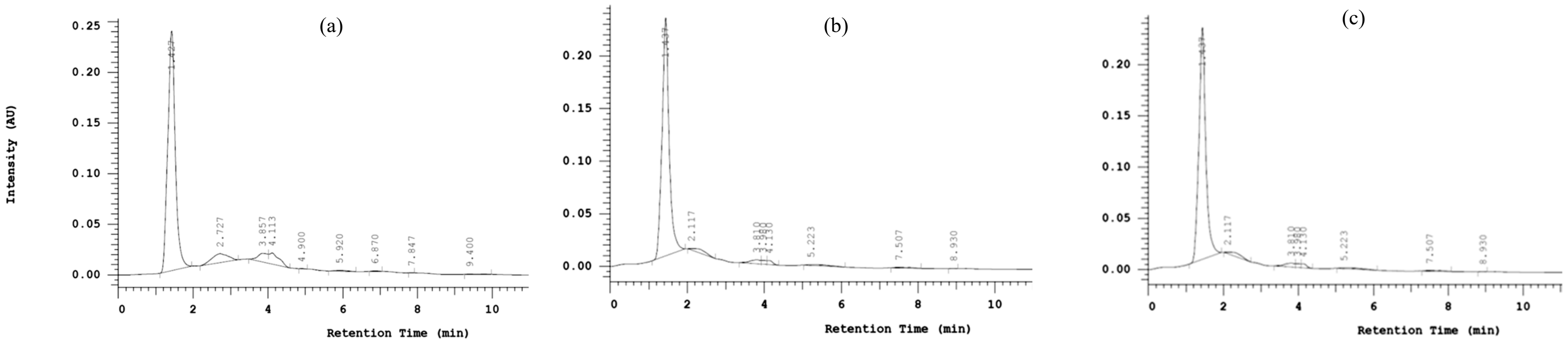

Figure S5 chromatogram of Mali Nil Surin growing in Bangkok (a), Trat (b) and Sakon Nakhon (c) province.

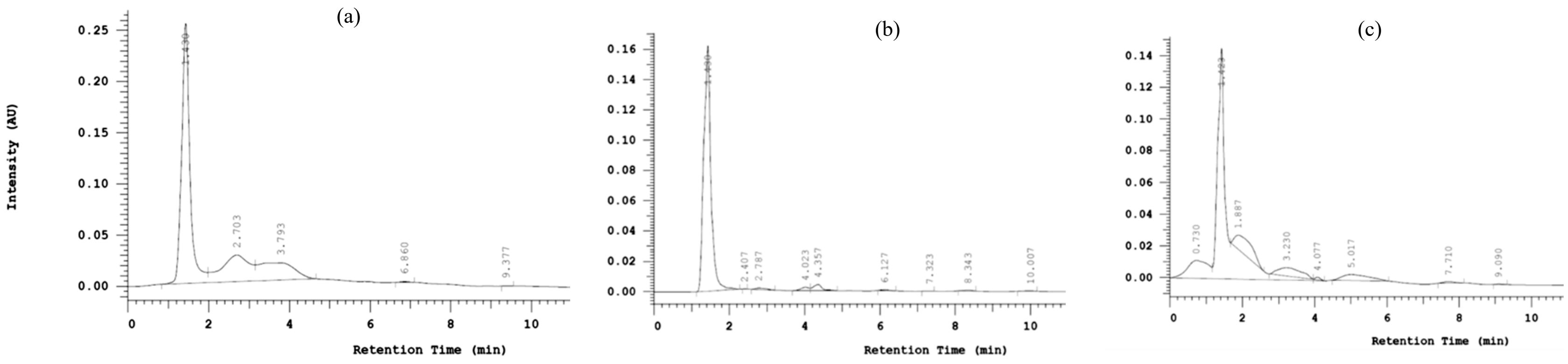

Figure S6 chromatogram of Riceberry growing in Bangkok (a), Trat (b) and Sakon Nakhon (c) province.

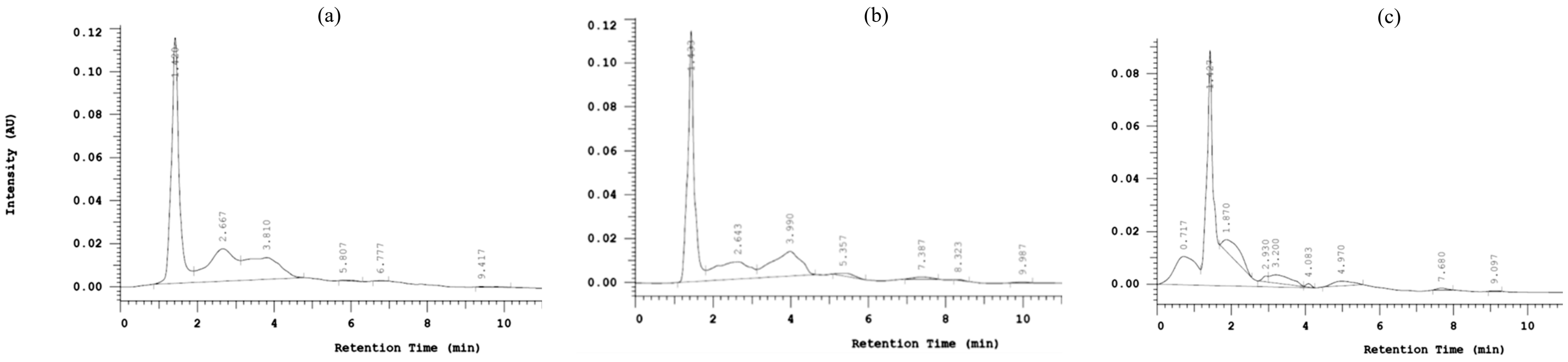

Figure S7 chromatogram of KDML105 growing in Bangkok (a), Trat (b) and Sakon Nakhon (c) province.
